# Dementia Caregivers’ Perspectives on Technology’s Place in Care Practices: Mixed Methods Survey

**DOI:** 10.2196/69596

**Published:** 2025-11-04

**Authors:** Julia A Scott, Emma Cepukenas, McKenzie Himes, Kennedy Anderson, Kiren Grewal, An Mai, Sheila Yuter, Patricia Simone

**Affiliations:** 1 Department of Bioengineering, Santa Clara University, 500 El Camino Real, Santa Clara, CA, 95053, United States, 1 (408) 554-4000; 2 Department of Public Health Sciences, Santa Clara University, Santa Clara, CA, United States; 3 Department of Biology, Santa Clara University, Santa Clara, CA, United States; 4 Department of Public Health Sciences, Santa Clara University, Santa Clara, CA, United States; 5 Department of Psychology, Santa Clara University, Santa Clara, CA, United States

**Keywords:** dementia, caregiving, technology, AgeTech, assistive technology, accessibility

## Abstract

**Background:**

As the number of individuals diagnosed with dementia continues to grow, collective caregiving capacity is simultaneously declining. As a result, caregivers often face significant stress and burnout, which negatively impacts both their well-being and the quality of care they are able to provide. To address these challenges, various forms of technology have been developed to support caregiving responsibilities for people with dementia. Barriers to widespread adoption include the cost of technology, lack of caregiver training, limited awareness of available solutions, privacy and ethical concerns, and resistance to change from both caregivers and care recipients. By identifying these key insights, this study seeks to inform the development of more accessible, user-friendly, and effective technological solutions that can better support caregivers in their vital roles.

**Objective:**

This study aimed to assess the way dementia caregivers solve common challenges, focusing on technology adoption and barriers to its use.

**Methods:**

We conducted online surveys regarding technology usage and caregiving experience, which were distributed to the communities of caregivers of people living with dementia. Surveys quantified categories of technology use and caregiver needs. The survey also gathered open responses for joys, struggles, and technology use. Follow-up interviews with caregivers discussed how specific challenges were met. The responses were thematically coded to characterize findings by the type—struggles, social supports, technology, and joy.

**Results:**

Respondents (N=69) identified access to resources as the most important unmet need for caregivers (53%). The survey found that medication and routine tracking applications and mobility assistive devices were the most commonly used forms of technology. The dominant need expressed by caregivers was for better access to resources, with improved access to health care services and financial support. Interviewees reported barriers to accessing resources, including high costs and difficulties in selecting appropriate technology resources. Additionally, centralizing resources and simplifying the process of locating support was recommended as a solution.

**Conclusions:**

The study showed that caregivers felt inadequately supported and did not frequently use technology due to barriers of access and usability of products. Strategies in product design and user communication that engage dementia care partners would likely accelerate adoption.

## Introduction

Caring for individuals with dementia is demanding and requires strong, effective systems that support the safety, well-being, and quality of life of both the person living with dementia (PLWD) and their caregivers. Most caregivers are informal, typically spouses, family members, or friends who take on the role without formal training or financial compensation [1,2]. A significant number of informal caregivers experience mortality at a higher rate than the person they are caring for, highlighting the immense strain and pressure associated with this role [3], and they often face a decline in their own health, frequently stemming from chronic stress, sleep disturbances, and the emotional toll of providing constant care and support [4]. Despite the well-documented negative effects on informal caregivers, these challenges continue to persist due to the inequitable distribution of social and health care support services [5,6]. Notably, mental health concerns represent the most frequently reported unmet need among caregivers, emphasizing the urgent requirement for interventions targeting psychological well-being [7]. Caregivers frequently express feelings of isolation and insufficient support from family, health care professionals, and employers, further underscoring the systemic gaps in caregiver assistance [8].

The burden faced by caregivers highlights the importance of targeted support. Studies have explored the unmet needs of informal caregivers using an online survey across various domains, including health information, self-care, and support service accessibility [9]. Their findings revealed a critical need for targeted support systems that focus on caregivers’ self-care, stress management, and communication skills. The study found that digital platforms could play a role in addressing these needs, given the potential for broad dissemination and accessibility. Such platforms have the potential to provide caregivers with on-demand access to educational resources, peer support networks, and mental health services, which could significantly alleviate caregiver burden.

Advancements in consumer technology have significantly transformed dementia care, providing essential support for both individuals with dementia and their caregivers. Wearable devices [10,11], interactive robots, mobile apps [12], and smart home systems are all emerging technologies that address the unique challenges faced in dementia care. There is also a web of offerings available to dementia caregivers. While these technologies offer promising support, challenges such as usability, cost, efficacy, and privacy concerns remain prevalent [13-15]. These are barriers to implementation in direct-to-consumer markets and health care systems. The reach of assistive technologies for caregivers is shallow because the system of care is not equipped to integrate the tools [16]. Addressing these issues is crucial for maximizing the benefits of technology in dementia care [13,14].

Projections for the technology used by older adults are growing. American Association for Retired Persons estimated that older adults would spend over US$120 billion on technology in 2023, reflecting both the growing digital engagement of this population and the increasing relevance of technology in health and social care contexts [17]. Concurrently, the global AgeTech market, which encompasses technological innovations designed to support aging populations, is valued at approximately US$45 trillion [17]. Within this expansive market, technology tailored for dementia care represents a rapidly expanding segment. Despite this growth and the global recognition that caregivers need support [2], widespread availability of dementia-related technologies is still emerging. Many technology services on the market are not yet scaled and are wholly in the direct-to-consumer market. The barriers previously stated have not yet been fully addressed.

Given these constraints, there is a critical need to align caregiver needs with appropriate technological interventions. A targeted needs assessment that considers the daily challenges faced by caregivers and evaluates the suitability and effectiveness of available digital tools is essential. When effectively implemented, such technologies hold the potential to reduce overall costs of care, facilitate aging in place, and alleviate caregiver burden, both in terms of workload and psychological stress.

One review reported on 31 studies published between 2007 and 2018 that address the needs of caregivers of PLWD; only a small number of these involved in-person interviews with caregivers, and many of these offered little insight on caregiver support [8]. Much of the existing data on caregiver needs comes from categorical surveys, which offer limited insight into why certain supports, like technology, are underused. However, a recent qualitative study with 12 informal dementia caregivers specifically asked caregivers about their use of technology [18]. They found that caregivers were open to using technology for practical tasks like scheduling, reminders, and information gathering, as well as video calls that support communication. However, they resisted using technology for emotionally meaningful tasks, such as bathing, cooking, or deep conversations, seeing these as essential to maintaining personal connection and dignity. The study emphasizes that technology should support, not replace, this “invisible emotion work.” Tools that aid reflection, evoke shared memories, or reinforce coping strategies (like exercise or peer support) can enhance caregiving without diminishing its human core.

The present study sought to assess the experiences of informal caregivers who provide care for PLWD. In contrast to a deficit-focused approach, this study also aimed to capture positive aspects of caregiving and the role of technology in both caregiving responsibilities and caregiver self-care. Data collection involved a mixed-methods approach. An anonymous survey collected quantitative and qualitative data on caregiving challenges, technology usage, and perceived needs. In addition to structured items, open-ended responses allowed caregivers to elaborate on their experiences. To further explore specific caregiving dilemmas and the strategies used to address them, in-depth follow-up interviews were conducted with a subset of participants.

This caregiver perspective study applies a balanced quantitative and qualitative approach to explore the lived experiences of dementia caregivers and their use of technology. By capturing rich, contextual narratives and a history of technology practices, the research will offer insights to guide the design of user-centered technologies that support both the functional and emotional needs of caregivers.

## Methods

### Ethical Considerations

The purpose of this study was to collect first-hand reports on experiences of dementia caregivers. The study was approved by the institutional review board for human subject research of the Santa Clara University. Informed consent was provided via a digital copy, which described the study purpose of identifying unmet needs of caregivers and potential discomfort in remembering aspects of caregiving. Participants were not compensated for completing the surveys or interviews. The study gathered data in two forms: (1) written surveys and (2) didactic interviews. The written surveys did not include identifying information and were coded by study ID. The interview transcripts were redacted for identifying information prior to analysis.

### Participants

The study included any person with caregiving experience (current or within 3 years) for Alzheimer’s and related dementias. Forwardable email invitations with links to anonymous surveys were distributed to the faculty, staff, and graduate students of Santa Clara University. The invitation was also distributed by the University of Washington Memory Hub in Seattle, Washington, and the Hearts and Minds Activity Center in San Jose, California. Within the survey, the option to participate in a video conference interview was included. Interview invitations were sent to known care professionals in the communities as well. The data was collected in April 2022.

### Surveys

An anonymous online survey (Multimedia Appendix 1) was developed by the research team to contribute to a formal needs assessment in the first step of a design project for dementia care innovation. Participants were asked demographic questions, about their caregiving history, to list their primary needs, support systems, and technology used. Next, they were prompted to describe joyful and difficult aspects of their caregiving experiences and technology needs.

### Interviews

Survey respondents (informal caregivers) and dementia care professionals (formal caregivers) were invited for interviews (Multimedia Appendix 1). Interview participants were asked about how they approached specific problems or challenges in caregiving practices. The structured interview followed a customer discovery method (Customer Development Labs), which probes how individuals approach meeting a specific need with nonleading questions and with an interactive cycle of questions, a method that is widely used for the exploration of needs assessment in user research. The purpose of this approach was to gather data for the subsequent phase of the project that aimed to develop new technologies for dementia care.

### Analysis

Survey responses were tabulated and segmented by key demographic variables--age of caregiver, role of caregiver, gender of caregiver, and years of caregiving experience.

The interview transcripts and survey narrative responses were analyzed by thematic coding, a qualitative research method used to identify, analyze, and report patterns (themes) within qualitative data [19,20]. Each entry was coded with up to three annotations for each item, organized within the themes of struggles, social supports, technology, and joy (Textbox 1). All responses were annotated by two raters. Any conflict in labeling selections was resolved by consensus via discussion between raters and the principal investigator.

Textbox 1. **Key topics annotated for thematic coding**

**Struggles**

Finances, Health care, Long-term care, Family responsibilities, Work performance, Emotional struggle/conflict, Personal health, Personal care of patient, Communication with patient, Daily routines, Isolation, Language/culture, Access to resources, Disorientation/recall, Safety

**Social supports**

Family, Eldercare staff, Support groups, Advocacy organizations, Friends/colleagues, Therapist/counselor, Social workers, No support

**Technology**

Trackers, Telehealth, Adaptive clothing, Mobility devices, Monitors/sensors, Media, Games, Smartphone apps, No tech helped

**Joys**

Remembrance, Family/social events, Spectator events/shows, Music/dance, Art, Travel, Rare expressions/speech, Expressions of gratitude/connection, Religious experiences, Being with children, Socializing with peers, Being in nature

Mixed methodology was applied by contingency tables. These tables were built for the narrative annotations by age of the caregiver, gender of the caregiver, age of the person affected with dementia, and relationship to the person affected with dementia, which provided context to the types of challenges and solutions expressed by the participants. Quotes were extracted and grouped from the responses to capture feelings and anecdotes not illustrated in the coded analysis. Collectively, these analyses synthesized the responses in a manner to infer the unmet needs of caregivers. This last step, which is not statistical, was conducted through consensus discussions between the authors.

## Results

### Survey Sample Demographic

The survey of informal caregivers (n=69) represented a range of caregiver roles (Table 1) . Nearly three-quarters of respondents were female and a similar proportion identified as White. Most of the caregivers surveyed were 55‐74 years of age; about 30% were younger and 9% were older. A quarter of caregivers took care of their spouses, and half cared for parents. All but one of the respondents cared for a person with dementia older than 50 years. Half of the respondents were currently caring for someone with dementia; of these, half had been in the role for 1 to 3 years, while the remainder had longer caregiving experiences. Twenty-three percent of the respondents have cared for more than one person with dementia.

**Table 1.** Demographic characteristics of the survey caregiver respondents (N=69).

Demographic characteristic	n (%)
Caregiver sex	
Male	50 (72.46)
Female	16 (23.19)
Non-binary	1 (1.45)
Caregiver race/ethnicity	
Asian	6 (8.70)
Black or African American	2 (2.90)
Hispanic (Non-White)	4 (5.80)
Middle Eastern	1 (1.45)
Samoan	1 (1.45)
White	51 (73.91)
White, Asian	1 (1.45)
White, Hispanic (Non-White)	2 (2.90)
White, Mexican-American (White)	1 (1.45)
Caregiver age (years)	
18‐25	2 (2.90)
26‐35	5 (7.25)
36‐55	15 (21.74)
55‐75	41 (59.42)
75‐95	6 (8.70)
PLWD^a^ age (years)	
<50	1 (1.45)
>75	38 (55.07)
50‐75	30 (43.48)

a PLWD: person living with dementia.

### Informal Caregiver Needs Assessment

**Figure 1.**
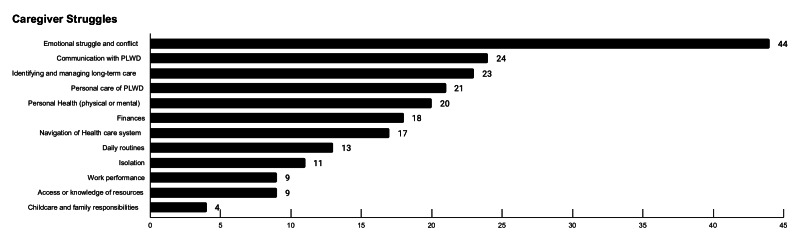
Ranked number of survey responses for caregiver struggles.

Participants listed the top three struggles in their caregiving role (Figure 1), offering a clear picture of the multidimensional challenges faced by informal caregivers of PLWD. Emotional struggle and interpersonal conflict emerged as the most frequently cited challenge, identified by 64% of respondents. Many caregivers described the emotional toll of witnessing cognitive decline and behavioral changes in their loved ones, often leading to frustration, grief, or feelings of helplessness. A resonating scenario of these challenges was shared here:

*My mother would sometimes walk out the front door and start walking to the neighborhood park on the next block. If it was too cold, I would struggle trying to get her back in the house. She was very stubborn and you can’t make a person with dementia do something she doesn’t want to do. They act like children many times and do not listen. Sometimes she would just sit on the sidewalk so I could not walk her back. If I tried to pick her up, she would scream for the police yelling that I was hurting her! It would be so embarrassing!*

In addition, 29% of participants reported that caregiving had negatively affected their physical or mental health. Caregivers frequently mentioned sleep disturbances, chronic stress, and isolation as ongoing struggles. A spouse caregiver illustrated this well: “I struggled with feeling trapped (eg, couldn’t get to a pool workout to take care of myself) because I couldn’t leave my husband alone.”

Care management issues also figured prominently in caregivers’ responses, with long-term care planning (33%), financial concerns (26%), and difficulties navigating the health care system (25%) cited as key stressors. These challenges reflect broader structural barriers to coordinated dementia care and indicate opportunities for targeted policy or programmatic interventions. Communication difficulties (35%) and assistance with personal care tasks (30%) were also frequently mentioned, reflecting the hands-on demands of caregiving and the evolving needs of PLWD as the disease progresses.

**Figure 2.**
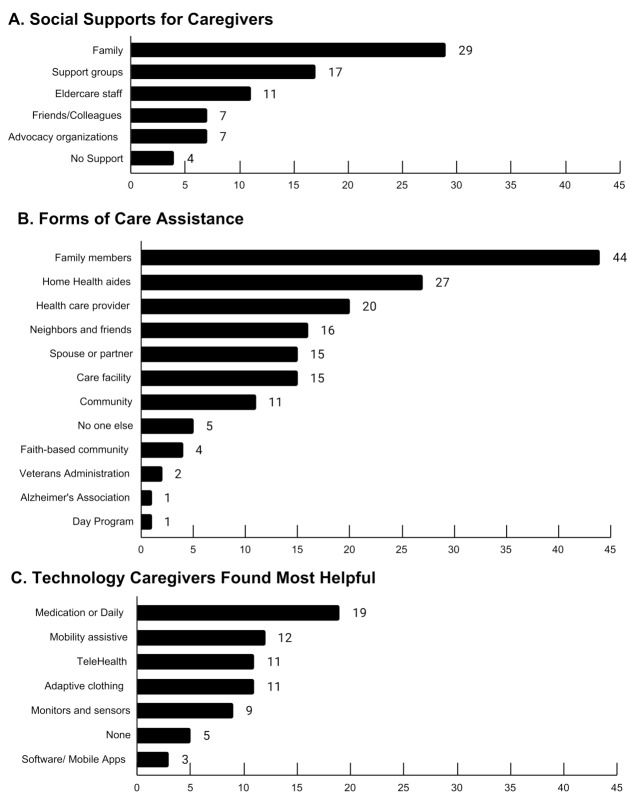
Ranked number of survey responses for (A) social supports, (B) assistance, and (C) technology use.

Support for caregivers was assessed across social, direct care, and technological domains (Figure 2). Family members were cited most often as sources of social support, with 42% identifying them as primary helpers. Support groups (25%) and paid care staff (16%) also played meaningful roles. Despite these reported connections, many caregivers noted the uneven or inconsistent nature of their support networks, which often left them feeling like they were “on call” around the clock. This further emphasizes the emotional isolation that can accompany informal caregiving. One adult child recounted how her parents would argue:

It was mentally draining because no matter how much I could deal with my step-father, my mother’s reactions would exacerbate the situation so it felt like I was dealing with two patients and not one. And the fact that my patience was beyond what I could possibly handle, left me feeling drained, frustrated and unempathetic toward them.

When it came to direct care, 64% of respondents received help from family members (other than a spouse), 39% shared caregiving duties with home health aides, and 29% received support from health care providers. While some described these supports as vital lifelines, others expressed frustration with the unpredictability or limited scope of external assistance.

Technology use among caregivers was relatively modest and task-specific. The most common tools were medication and daily tracking apps (28%), used to manage schedules and routines. Over 15% of participants reported using tools that offered practical, hands-on support such as mobility aids, telehealth platforms, or adaptive clothing designed for people with physical disabilities or limited mobility, featuring easy closures, adjustable elements, and comfortable designs for independence. However, more advanced or integrated technologies, such as home monitors, sensors, or caregiving-specific platforms, were used by only 13% of respondents. Notably, none of the participants mentioned using technology for emotional or relational support.

Overall, these findings underscore the complexity of the caregiving experience and highlight important opportunities for designing tools and services that better reflect caregivers’ lived realities, particularly by recognizing and supporting the emotional labor involved in their roles.

### Survey Narrative

**Figure 3.**
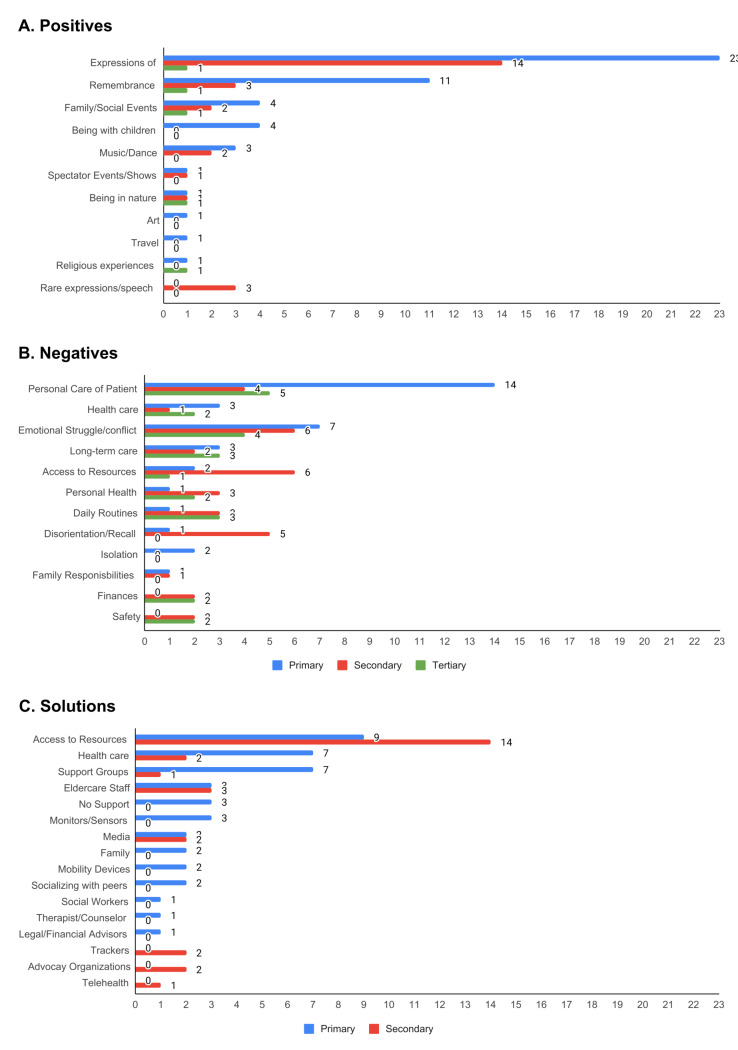
Occurrence of annotations for (A) positives, (B) negatives, and (C) solutions, survey narrative response sets. Bars represent total counts of each categorization. If multiple annotations were found in a response, they were ranked from primary to tertiary.

Open-ended responses about caregiving joys (n=55), struggles (n=56), and unmet needs or solution gaps (n=53) were annotated for themes, revealing distinct patterns in caregiver experiences (Figure 3). These qualitative insights, detailed in Multimedia Appendix 1, provide important nuance to the survey data and help illuminate the emotional and relational dimensions of caregiving that are often underrepresented in categorical data.

The most frequent positive experiences cited were grounded in interpersonal connection. Caregivers described moments of expressed gratitude or emotional closeness (72%) as the most meaningful aspects of their role. Instances of remembrance and reminiscence (29%), family or social gatherings (13%), and time spent with children (8%) were also commonly named. These patterns were consistent across gender, caregiver age, and relationship to the PLWD, emphasizing the universality of emotional bonding in caregiving. Even when the focus was on remaining abilities and bonding, there was often bittersweet undertones, as described by an adult child:

*She’s my mother and accepting of her dementia, a positive person and appreciative. She is also a historian, so since I retired I have had more time to help with this task. It makes me sad how much info I will lose when she is gone, and she drives me nuts with her efforts, and sad with her difficulties. Still I value the time.*

Narratives describing caregiving struggles echoed and deepened the findings from the structured survey. Caregivers frequently cited hands-on personal care, communication difficulties, and emotional strain or conflict as primary challenges. These narratives often conveyed a sense of emotional exhaustion, confusion, or sadness, especially when trying to interpret or respond to changed behaviors in the PLWD. Male caregivers were more likely to report health care system navigation and resource access as their central concerns (17% and 25%, respectively), compared to female caregivers (5% and 12%). Notably, challenges related to personal care were more frequently reported when the PLWD was a mother or mother-in-law (45%) or grandparent (40%), suggesting that gendered norms of caregiving or discomfort with certain caregiving tasks may play a role in perceived burden. A husband describes his biggest pain point in the area of health care:

*My spouse’s refusal to cooperate with physical therapy, occupational therapy and transition to mobility assistance devices.*

When asked about unmet needs, caregivers overwhelmingly emphasized the lack of access to practical resources (53%), such as information, services, or structured assistance. Health care service needs (25%), financial advising or aid (21%), and support group availability (17%) followed. Interestingly, only female respondents mentioned a desire for financial support, and younger caregivers (ages 36‐55 years) were nearly twice as likely to name financial challenges (33%) compared to older caregivers (17%). These findings point to gender and generational differences in financial strain or planning expectations and may reflect different stages of life or career interruption due to caregiving demands.

Overall, the narratives underscore a dual reality of caregiving: caregivers deeply value emotional connection and shared meaning with the PLWD, yet they are often left without adequate support to manage the demands that threaten those very connections. These findings reinforce the call for technology and service solutions that respect both the logistical and emotional realities of caregiving: tools that can streamline care tasks without undermining the relational work caregivers view as core to their identity and role.

### Interviews

#### Overview

The interviews were conducted with both formal and informal caregivers to gain a deeper understanding of caregivers’ lived experiences and identify strategies to improve dementia care and alleviate caregiver burden—insights that extend beyond what could be captured in the survey. Through these conversations, the study aimed to inform the development of new tools, services, or supports that are directly responsive to caregiver needs. Summary and annotations for each interviewee are documented in Multimedia Appendix 1.

Five interviewers conducted in-depth interviews with 18 caregivers. Of these, 78% were female and 22% male, and the majority (75%) identified as white. The participants included 16 informal caregivers (7 spousal caregivers, 5 adult children caring for a parent, and 4 extended family caregivers) and one each from the perspectives of a therapist/specialist, nurse, and activity center staff (also counted in adult child group). Eleven of the informal caregiver interviewees had also completed the survey, allowing for a more layered understanding of their caregiving experiences. Detailed synopses and annotated transcripts are available in the Supplemental Materials in Multimedia Appendix 1.

Analysis of the interview data revealed several recurring categories of concern and support. The most frequently mentioned pain points included emotional struggle, communication challenges with the PLWD, and long-term care planning, all of which mirror the findings from the broader survey. At the same time, caregivers identified family members, support groups, and paid care staff as their most helpful resources. Positive experiences often centered on expressions of gratitude, emotional connection, and reminiscence by the PLWD, which caregivers described as small but meaningful affirmations of their role. Conversely, frustrations were rooted in the physical demands of personal care, the stress of financial management, and the persistent emotional toll of caregiving.

When asked about potential solutions, caregivers overwhelmingly called for greater access to reliable resources, more support group opportunities, improved health care navigation, and direct financial assistance. These priorities reflect a broader need not just for services but for systems that acknowledge and respond to the complex, emotional, and practical nature of dementia caregiving. For example, an adult child who also worked in a memory care center expressed how grief for her mother was a daily battle. She leaned heavily on her coworkers for emotional and practical support.

Notably, the 30 direct quotes coded from the interviews reveal where caregivers placed their emphasis: 57% (17 quotes) reflected concerns about personal well-being, including stress, burnout, and the need for time away. Twenty-three percent (7 quotes) expressed hopes and desires for better systems, including tools to reduce isolation or enable earlier planning. The remaining 20% (6 quotes) focused on strategies and potential solutions, such as improved information flow between providers, streamlined financial support systems, and technologies that could promote connection without displacing caregiver control.

Together, the interview findings provide a deeper, more human perspective on the challenges and joys of caregiving. They reinforce the importance of designing solutions that support caregivers both emotionally and practically and that recognize caregiving as not just a role, but a relationship grounded in meaning, memory, and love.

#### Well-Being

Caregivers frequently described their personal well-being as marked by frustration, exhaustion, and loneliness, reflecting patterns well documented in the caregiving literature [4,7,13]. Repetition and monotony were recurring themes as one participant described feeling like “an actor in a play with the long run,” while another shared, “I’m having basically the same conversation over and over again.” Feelings of isolation were also strong, with one caregiver stating, “You are alone for so long.” Many described being emotionally and mentally drained, with some acknowledging they were neglecting their own physical health in favor of caregiving responsibilities. One caregiver admitted, “I don’t think I’m taking good physical care of myself.” Others spoke of the emotional toll of facing dementia’s progression, describing it as “a harsh reality” and difficult to accept.

#### Hopes and Desires

Caregivers expressed clear hopes for better educational resources, financial support, and stronger communication networks throughout the caregiving journey. Several participants emphasized the need for more accessible information about available services, with one noting the absence of “safety nets for seniors” and the limited support for low-income families. Others highlighted a desire for professional guidance, such as access to a “financial advisor, tax advisor, or pro bono attorney,” to help navigate the complex financial aspects of caregiving. Communication gaps with care facilities were another frustration, with caregivers reporting delays or difficulty reaching staff.

#### Challenges

Interviewees proposed a range of solutions to ease caregiving challenges, including wellness programs for care partners, supportive community platforms, and digital tools to streamline caregiving tasks. Suggestions included exercise and mindfulness activities, like yoga, which one caregiver used in a senior day center to support both client function and caregiver–client connection. Others highlighted the value of indirect peer support, such as podcasts or articles, which helped them feel less alone and more informed. Several participants emphasized the burden of financial strain, noting that resources like a “Medicare workshop” or access to financial advisors could be valuable. On the tech front, one caregiver expressed interest in a centralized app to manage daily activities, bookmark helpful sites, and stay organized—underscoring the need for tools that enhance, rather than complicate, the caregiving experience.

## Discussion

### Principal Results

This study examined the concerns of and strategies taken by informal caregivers of PLWD in metropolitan, tech centers of the west coast regions of the United States. It showed that current use of technology provides limited support for the needs of caregivers. The majority of respondents were informal caregivers, and the most common concerns were related to the personal care of the PLWD, communication with the PLWD, and emotional struggle and conflict. The main source of social support for informal caregivers was identified as family, followed by support groups and care staff. Caregivers commonly reported using technology for medication and daily tracking, with other popular tools being mobility assistive devices, telehealth services, and adaptive clothing. A small number of respondents used monitors and sensors. The dominant need expressed by caregivers was for better access to resources, and improved access to health care services, financial support, and support groups was also frequently mentioned. These insights echo broader findings that caregivers need not only emotional and practical support but also clearer pathways to resources and systems that respond more efficiently to their evolving needs [8,16,21].

The interviews provided clear evidence of how dementia impacts not only the individual living with the condition but also their caregiver. Caregivers primarily focused on the effects on their personal well-being that led to loneliness, frustration, helplessness, and exhaustion. To alleviate their burden, several caregivers suggested improving resource accessibility. These respondents were aware that many support mechanisms existed, including online resources, support groups, and health care services, and acknowledged that specific strategies can ease their workload and alleviate negative emotions associated with caregiving. However, barriers to accessing resources were commonly cited, including high costs and difficulties in selecting appropriate resources. Additionally, centralizing resources and simplifying the process of locating support was recommended as a solution. Digital technologies, if made affordable and easy to use, could help in these services.

### Comparison to Prior Work

The results of this study align closely with previous reports on the needs of dementia caregivers. Research consistently shows that caregivers experience higher levels of stress, depression, and anxiety symptoms compared to noncaregivers [4,7]. Previous studies have identified primary stressors such as emotional outbursts or behavioral problems from PLWD as the strongest predictors of poor health outcomes among caregivers [7,22]. Our survey and narrative data reinforce that emotional struggle and interpersonal conflict with the PLWD are the primary concerns among most caregivers. However, the majority of existing data on caregiver needs has been gathered through categorical surveys and fails to provide sufficient detail on specific caregiver pain points. This report complements larger-scale surveys, such as the annual *Alzheimer’s Association Facts and Figures* report [4], by offering qualitative depth and contextual understanding.

Moreover, a wide range of educational and psychosocial interventions have been shown to benefit caregivers within comprehensive care models [4,22]. Specifically, interventions that are personalized and actively involve family members tend to be more effective [7,8,18,23]. In addition to mitigating negative outcomes, supporting meaningful activities between care partners can have a substantial impact on caregiver well-being. Caregivers who report more positive interactions with the PLWD, such as moments of gratitude or shared remembrance, demonstrate better mental health outcomes [24]. Our survey illustrated this relationship: caregivers who reported emotionally positive experiences were less likely to indicate poor personal health and psychological distress. Although digital technologies like DevaWorld and FamilyShare have emerged to promote connection and engagement, none of our participants reported using such tools, suggesting a gap between available resources and actual caregiver uptake.

Access to resources also emerged as the most commonly cited unmet need, consistent with prior research highlighting caregivers’ desire for more support from health care providers, family, and community services [8]. Research is growing to inquire on which technologies caregivers are currently using or how they envision technology helping them meet their needs, especially for mobile applications [12,25]. This underscores a need for more caregiver-centered inquiry. For example, Smriti et al identified the ongoing management of feelings and emotional connection as a central aspect of dementia caregiving [18]. Their study found that while caregivers were willing to use technology for practical tasks (eg, reminders, telehealth), they resisted using it for emotionally meaningful caregiving activities, such as bathing or deep conversations. These emotionally rich tasks are viewed as essential to caregivers’ identities and bonds with the PLWD. Smriti et al emphasized that technology design should not aim to replace this emotion work but rather support it through features that foster reflection, shared memories, or emotional resilience. Our findings align with this approach: caregivers valued tools that reduced practical burdens but wanted to retain control over emotionally significant aspects of caregiving. As digital solutions continue to evolve, developers and care systems must adopt a holistic, caregiver-informed approach to technology design that supports, not supplants, the human core of caregiving.

### Technology Adoption Recommendations

#### Overview

Dementia is already one of the most pressing issues affecting our aging population, currently affecting around 50 million people worldwide, and this number is expected to triple by 2050, meaning the demand for caregivers will rise dramatically in stride [8]. The cost of dementia care to society will compound by the additional impact on caregivers’ physical and mental health. Persistent high workload and emotional strain on caregivers increase the likelihood of transitioning from aging-in-place to residential care [26] and are associated with increased medical needs of the caregiver [4]. The summative effect of a fractured model of dementia care is ultimately more expensive and delivers a poorer quality of care, compared to comprehensive care models [21,27,28]. Assistive technology can play a significant role in improving the caregiving experience for both the caregivers and the individuals receiving care [1,13,29]. As new technologies emerge, it is critical to focus on innovation that can address the specific needs of caregivers. One approach to this is summarized by Kiselica et al, which draws a framework to apply common technologies to dementia care [30].

In response to this, many organizations are currently working to support innovation and provide solutions to address the specific needs of people affected by dementia. For example, the American Association for Retired Persons has teamed with the Alzheimer’s Association, Challenge Works, and other organizations in sponsoring an international competition to find innovative solutions for dementia care [31]. The competition focuses on technology-based solutions such as using artificial intelligence to provide better care to PLWD while reducing caregiver workload. The Alzheimer’s Association has also launched an initiative called the Alzheimer’s Association Innovation Roundtable, which sets the goal to strengthen existing approaches while developing new technology that reimagines dementia care. The roundtable offers impact-focused meetings, webinars, and an annual global innovation competition called the Alzheimer’s Association Pitch Competition [32].

It is important to address that most caregivers are not using technology to address their highest areas of concern, such as locating support resources and dealing with emotions of frustration and helplessness. Even in our sample from “Silicon Valley” and Seattle, where digital technology innovation is the economic engine, technology solutions are not reaching the daily lives of caregivers. It would be fair to extrapolate that in areas that are lower resourced and with sparser internet connectivity, the adoption rates would be even lower. There are several factors that contribute to caregivers’ current underutilization of technology, such as the challenge of identifying appropriate technology solutions, the high costs of existing products, and the scarcity of solutions specifically tailored to the unique needs of dementia care. The following are recommendations based on the caregiver responses that could improve adoption of technology for dementia care.

#### Need for Centralized Resources

Despite the large number of resources in existence for caregivers, study respondents noted that it is often difficult to sort through and find relevant information, such as difficulty finding a local support group in large databases or through a search engine. Therefore, it is critical to think about centralizing resources in a manner that facilitates easy navigation for users. My ALZ Journey mobile app was released by the Alzheimer’s Association in 2025 to address navigation from diagnosis to long-term care plans [33]. The reach of the organization is comprehensively national, and the app is available at no cost and does not store user personal information. The potential for this as an effective, centralized resource is clear. Whether it is utilized as intended will be observed with time.

#### Accessibility and Fit

Even after overcoming the challenge of identifying a product or service, additional barriers still inhibit usage. They include the complexity of user interfaces, the bias of English-only text, or recurring subscription fees. The prerequisite requirements to reach a broad population of caregivers are usability, cultural inclusivity, and affordability. Engagement of diverse stakeholders in product development and the investment in innovation for smaller populations will lead to improved accessibility and fit of digital tools for family caregivers. At present, a one-size-fits-all approach is evident in first-to-market solutions from established organizations (eg, My ALZ journey). The foundation of these applications can serve as the scaffolding for culturally aligned content.

#### Develop Stakeholder-Informed Solutions

Safety and monitoring assistive technologies are examples of how technology can improve the quality of life in both home settings and care facilities [16,34]. Video monitors and GPS devices to track the PLWD in real time have been shown to increase the perceived security of the PLWD for caregivers [35,36]. Smart-home systems have also gained attention for the ability to extend the PLWD with increased autonomy to live at home and ease caregiving responsibilities [37]. For example, automatic sensors, typically paired to a central control panel or app, can manage lights and doors and detect sudden heat changes. Additionally, web-based technology such as social network platforms, informative websites, and educational videos has been received positively by caregivers to increase their social support access to resources [38,39]. Collectively, these systems reduce the workload of caregivers while giving caregivers relevant information to deliver care.

### Limitations

There are several limitations to consider regarding the sample profile of this needs assessment study. First, nearly three-quarters of respondents were identified as White, which raises concerns that the results may not accurately reflect the experiences of caregivers from different cultural, educational, and socioeconomic backgrounds. Additionally, most of the caregivers in this study were 55‐74 years of age. This is higher than the average age of caregivers in the United States of about 50 years [40], which means that there may be differences in the unmet needs of individuals in this study by comparison to most studies. Specifically, technology usage is strongly influenced by age [41]. Thus, the low usage of technology-driven strategies and difficulty in finding resources digitally and via the internet, found in our sample, may only be representative of older adults, and accessibility may have improved since the time of this study in 2022. Future research should aim to include a more diverse sample and consider these factors to provide a broader understanding of the experiences of caregivers in different populations.

### Conclusions

The findings from this inquiry into caregivers of PLWD illustrate the complexity of their needs and the nuanced role that technology can play in alleviating caregiver burden. Through a mixed-methods approach that integrated both quantitative and narrative data, this study revealed that the greatest barriers to technology adoption are not a lack of available tools but rather limited accessibility, awareness, and fit with caregivers’ lived experiences. These barriers are particularly pressing given the growing interest in AgeTech and digital health solutions. Encouragingly, such barriers are more tractable than systemic resource shortages and can be addressed through coordinated action among dementia care advocates, public health institutions, and designers committed to inclusive, human-centered technologies.

Importantly, this study contributes to a deeper understanding of why caregivers often do not adopt existing technologies. As also emphasized in a recent scoping review, informational gaps and caregivers’ emotional and physical health remain among the most frequently cited unmet needs, especially when caregivers feel unsupported or lack time for self-care [8]. Emotional health was the most commonly reported need across the majority of the studies in that review, followed by a need for formal or informal help and better access to information. Our findings reinforce these trends and emphasize that interventions, whether digital or community-based, must go beyond utilitarian functions and instead support the emotional labor inherent in caregiving.

Technology, when properly designed, can help caregivers offload logistical tasks such as scheduling, tracking medications, and communication but should avoid replacing emotionally meaningful interactions that define caregiving identity and purpose. As other researchers have noted, attempts to fully automate emotional aspects of caregiving risk undermining what caregivers value most about their role [19]. Instead, digital tools should aim to augment emotion work by enabling reflection, shared memory, and just-in-time support.

Ultimately, this study highlights the urgent need for cross-sector collaboration among health professionals, caregivers, researchers, technologists, and policymakers to develop resources that are accessible, context-sensitive, and culturally appropriate. Addressing caregivers’ unmet needs will require not just innovation but integration: aligning digital tools with psychosocial supports, community networks, and education tailored to different stages of dementia. In doing so, we can better support caregivers in sustaining both their caregiving responsibilities and their own well-being.

## Supplementary material

10.2196/69596 Multimedia Appendix 1 Summary and annotations for survey and interview datasets.

## Abbreviations

PLWD: Persons living with dementia
