# Supplementary material for: Dementia Caregivers’ Perspectives on Technology’s Place in Care Practices: Mixed Methods Survey
Source: JMIR Form Res. 2025 Nov 4;9:e69596. doi: 10.2196/69596 (PMC12584996; doi:10.2196/69596)
Supplement: Multimedia Appendix 1 [file formative-v9-e69596-s001.pdf]

Appendix 1. Survey Questions

1. At SCU my position is ___.
    a. student
    b. faculty
    c. staff
    d. not-affliated with SCU
2. My age is ___ years.
    a. 18-25
    b. 26-35
    c. 36-55
    d. 55-75
    e. 75-95
3. I identify as ____.
    a. Male
    b. Female
    c. Non-binary
4. I identify as ______. (Check all that apply)
    a. White
    b. Black or African Americaon
    c. Hispanic (Non-white)
    d. Asian
    e. Other _________
5. I have cared for the following family relations with dementia
    a. Spouse
    b. Mother/Mother-in-law
    c. Father/Father-in-law
    d. Grandparent
    e. Aunt or Uncle
    f. Family Friend or Community Member
    g. Client
    h. None
    i. Other _________

If more than one check box is marked, please respond to following questions in regard to your most involved caregiving role.

6. I am currently a caregiver of a person with dementia.
    a. Yes
    b. No
7. The person I cared for was ____ years old at the time I started.

    a. <50
    b. 50-75
    c. >75
8. I cared for this person ____ years ago.
    a. <1
    b. 1-3
    c. 4-10
    d. >10
9. I was assisted in my caregiving responsibilities by ___. (Check all that apply)
    a. Spouse or Partner
    b. Family members
    c. Community organization
    d. Faith-based community
    e. Neighbors and Friends
    f. Vetrans Administration
    g. Home health aides
    h. Support staff
    i. Care facility
    j. The patient's healthcare provider
    k. No one else
    l. Other __________
10. The top three (3) areas I struggled with were ___.
    a. Finances
    b. Navigation of healthcare system
    c. Identifying and managing longterm care
    d. Childcare and family responsibilities
    e. Work performance
    f. Emotional struggle and conflict
    g. Personal health (physical or mental)
    h. Personal care of patient
    i. Communication with patient
    j. Maintaining daily routines
    k. Isolation
    l. Language and/or cultural barriers
    m. Access to or knowledge of resources
    n. Other _________
11. What social supports did you find the most helpful during your caregiving? (Check all that apply)
    a. Support groups
    b. Alzheimer's Association or other advocacy organizations

    c. Eldercare staff
    d. Family
    e. Other _________
12. What types of technological tools did you find most helpful? (Check all that apply)
    a. Medication or Daily trackers
    b. Telehealth
    c. Adaptive clothing
    d. Mobility assistive devices
    e. Monitors and sensors
    f. Other _________

In your own words, please share your experience of caring for someone with dementia. Avoid using any identifying information in your responses (e.g. Grandma's name, city names, hospital names, etc.). Please keep your responses within 250 words each.

13. Please tell us about a time you found joy in caring for someone with dementia. A retelling of a specific memory would really illustrate this well.
14. Please tell us about about a time you really struggled in your caregiving role. Reflecting on an event where you felt you didn't have the answers or a solution would get to the heart of this question.
15. What type of help or tools did you wish you had at the time of caregiving but simply did not exist or were not accessible?

Appendix 2. Interview Script

Structured interview questions.

1. What's the biggest challenge you're facing as a caregiver?
2. When was the last time you tried to solve that problem?
3. Can you tell me about the last time that problem happened?
4. Why is it a problem for you?
5. How did you find your current solution?
6. What isn’t ideal about your current solution?

Optional follow up questions.

1. What's the another major challenge you're facing as a caregiver with respect to taking care of individuals with dementia?
2. I'm actually exploring solutions in dementia care. Can I contact you at our next stage of idea generation?
3. I’m trying to understand this problem from a wide range of perspectives.
4. Do you know 1 or 2 other people who are struggling with caregiving that I might talk with?

Appendix 3. Survey Narrative Analysis

Table 1. Coding of Survey Narratives by Gender

	Primary Positives				Secondary Positives				Tertiary Positives			
Tags	Female	Male	Nonbinary	Total	Female	Male	Nonbinary	Total	Female	Male	Nonbinary	Total
Expressions of gratitude/connection	48%	25%	33%	42%	39%	63%	0%	44%	13%	0%	0%	11%
Remembrance	20%	25%	0%	20%	0%	0%	0%	0%	0%	0%	0%	0%
Family/Social Events	5%	8%	33%	7%	9%	0%	0%	6%	13%	0%	0%	11%
Being with Children	8%	8%	0%	7%	0%	0%	0%	0%	0%	0%	0%	0%
Music/Dance	8%	0%	0%	5%	9%	0%	0%	6%	0%	0%	0%	0%
Daily Routines	5%	8%	0%	5%	4%	13%	0%	6%	38%	100%	0%	44%
Healthcare	0%	8%	33%	4%	0%	0%	0%	0%	0%	0%	0%	0%
Travel	0%	8%	0%	2%	0%	0%	0%	0%	0%	0%	0%	0%
Spectator Events Shows	0%	8%	0%	2%	0%	0%	100%	3%	0%	0%	0%	0%
Religious Experiences	3%	0%	0%	2%	13%	0%	0%	9%	13%	0%	0%	11%
Being in Nature	3%	0%	0%	2%	4%	0%	0%	3%	13%	0%	0%	11%
Art	3%	0%	0%	2%	0%	0%	0%	0%	0%	0%	0%	0%
Rare expressions/speech	0%	0%	0%	0%	9%	13%	0%	9%	0%	0%	0%	0%
Personal Care of Patient	0%	0%	0%	0%	4%	13%	0%	6%	0%	0%	0%	0%
Eldercare Staff	0%	0%	0%	0%	4%	0%	0%	3%	0%	0%	0%	0%
Disorientation/Recall	0%	0%	0%	0%	4%	0%	0%	3%	0%	0%	0%	0%
Total	40	12	3	55	23	8	1	32	8	1	0	9

	Primary Negatives				Secondary Negatives				Tertiary Negatives			
Tags	Female	Male	Nonbinary	Total	Female	Male	Nonbinary	Total	Female	Male	Nonbinary	Total
Personal Care of Patient	27%	25%	0%	25%	11%	14%	0%	11%	6%	0%	0%	5%
Communication with Patient	17%	25%	33%	20%	14%	14%	50%	16%	0%	20%	0%	5%
Emotional Struggle/conflict	17%	8%	33%	16%	14%	29%	50%	19%	25%	0%	0%	19%
Eldercare Staff	7%	0%	33%	7%	4%	0%	0%	3%	13%	20%	0%	14%
Healthcare	5%	17%	0%	7%	4%	0%	0%	3%	6%	0%	0%	5%
Isolation	7%	0%	0%	5%	0%	0%	0%	0%	0%	0%	0%	0%
Personal Health	5%	8%	0%	5%	0%	0%	0%	0%	13%	0%	0%	10%
Access to Resources	2%	8%	0%	4%	11%	29%	0%	14%	0%	0%	0%	0%
Safety	2%	8%	0%	4%	4%	0%	0%	3%	13%	0%	0%	10%
Disorientation/Recall	2%	0%	0%	2%	11%	0%	0%	8%	0%	0%	0%	0%
Family	2%	0%	0%	2%	4%	0%	0%	3%	0%	0%	0%	0%

Family Responsibilities	2%	0%	0%	2%	0%	0%	0%	0%	6%	0%	0%	5%
Longterm Care	2%	0%	0%	2%	11%	0%	0%	8%	6%	0%	0%	5%
Daily Routines	0%	0%	0%	0%	4%	14%	0%	5%	0%	20%	0%	5%
Finances	0%	0%	0%	0%	7%	0%	0%	5%	6%	40%	0%	14%
Work Performance	0%	0%	0%	0%	4%	0%	0%	3%	0%	0%	0%	0%
Access to Resources	0%	0%	0%	0%	0%	0%	0%	0%	6%	0%	0%	5%
Total	41	12	3	56	28	7	2	37	16	5	0	21

	Primary Solutions				Secondary Solutions				Tertiary Solutions			
Tags	Female	Male	Nonbinary	Total	Female	Male	Nonbinary	Total	Female	Male	Nonbinary	Total
Access to Resources	20%	0%	0%	15%	30%	57%	50%	36%	25%	0%	0%	23%
Healthcare	7%	40%	0%	13%	7%	0%	0%	5%	20%	0%	0%	18%
Support Groups	12%	20%	0%	13%	3%	0%	0%	3%	0%	0%	100%	5%
Finances	15%	0%	0%	11%	17%	0%	0%	13%	0%	0%	0%	0%
Communication with Patient	2%	10%	50%	6%	0%	0%	0%	0%	0%	0%	0%	0%
Eldercare Staff	7%	0%	0%	6%	7%	14%	0%	8%	0%	0%	0%	0%
Monitors/Sensors	7%	0%	0%	6%	3%	0%	0%	3%	0%	0%	0%	0%
No Support	7%	0%	0%	6%	0%	0%	0%	0%	0%	0%	0%	0%
Family	2%	10%	0%	4%	0%	0%	0%	0%	0%	0%	0%	0%
Media	2%	10%	0%	4%	3%	0%	50%	5%	0%	0%	0%	0%
Mobility Devices	5%	0%	0%	4%	0%	0%	0%	0%	0%	0%	0%	0%
Socializing with Peers	2%	0%	50%	4%	0%	0%	0%	0%	0%	0%	0%	0%
Legal/Financial Advisors	2%	0%	0%	2%	0%	0%	0%	0%	5%	0%	0%	5%
Longterm Care	2%	0%	0%	2%	10%	0%	0%	8%	10%	0%	0%	9%
Social Workers	2%	0%	0%	2%	0%	0%	0%	0%	0%	0%	0%	0%
Tech not Helpful	0%	10%	0%	2%	0%	0%	0%	0%	0%	0%	0%	0%
Therapist/Counselor	2%	0%	0%	2%	0%	0%	0%	0%	0%	100%	0%	5%
Advocacy Organizations	0%	0%	0%	0%	3%	14%	0%	5%	5%	0%	0%	5%
Trackers	0%	0%	0%	0%	7%	0%	0%	5%	0%	0%	0%	0%
Emotional Struggle/conflict	0%	0%	0%	0%	0%	14%	0%	3%	0%	0%	0%	0%
Personal Care of Patient	0%	0%	0%	0%	3%	0%	0%	3%	5%	0%	0%	5%
Telehealth	0%	0%	0%	0%	3%	0%	0%	3%	5%	0%	0%	5%
Personal Health	0%	0%	0%	0%	3%	0%	0%	3%	5%	0%	0%	5%
Daily Routines	0%	0%	0%	0%	0%	0%	0%	0%	10%	0%	0%	9%
Religious Experiences	0%	0%	0%	0%	0%	0%	0%	0%	5%	0%	0%	5%
Safety	0%	0%	0%	0%	0%	0%	0%	0%	5%	0%	0%	5%

Total	41	10	2	53	30	7	2	39	20	1	1	22

Table 2. Coding of Survey Narratives by Age Group

	Primary Positives					Secondary Positives					Tertiary Positives				
	The person I cared for was _____ at the time I started					The person I cared for was _____ at the time I started					The person I cared for was _____ at the time I started				
**Tags**	**26-35**	**36-55**	**55-75**	**75-95**	**Total**	**26-35**	**36-55**	**55-75**	**75-95**	**Total**	**26-35**	**36-55**	**55-75**	**75-95**	**Total**
Expressions of Gratitude/Connection	33%	46%	43%	25%	42%	33%	63%	42%	0%	44%	0%	0%	17%	0%	11%
Rememberance	33%	15%	20%	25%	20%	0%	13%	11%	0%	9%	100%	0%	0%	0%	11%
Being with Children	0%	15%	6%	0%	7%	0%	0%	0%	0%	0%	0%	0%	0%	0%	0%
Family/Social Events	0%	0%	11%	0%	7%	33%	0%	5%	0%	6%	0%	0%	17%	0%	11%
Daily Routines	0%	0%	9%	0%	5%	0%	0%	11%	0%	6%	0%	50%	33%	0%	33%
Music/Dance	0%	15%	3%	0%	5%	0%	13%	5%	0%	6%	0%	0%	0%	0%	0%
Healthcare	0%	0%	3%	25%	4%	0%	0%	0%	0%	0%	0%	0%	0%	0%	0%
Art	33%	0%	0%	0%	2%	0%	0%	0%	0%	0%	0%	0%	0%	0%	0%
Being in Nature	0%	0%	3%	0%	2%	0%	0%	0%	0%	0%	0%	0%	0%	0%	0%
Religious Experiences	0%	8%	0%	0%	2%	0%	0%	0%	0%	0%	0%	50%	17%	0%	22%
Spectator Events/Shows	0%	0%	3%	0%	2%	33%	0%	0%	0%	3%	0%	0%	0%	0%	0%
Travel	0%	0%	0%	25%	2%	0%	0%	5%	0%	3%	0%	0%	17%	0%	11%
Rare Expressions/Speech	0%	0%	0%	0%	0%	0%	0%	5%	100%	9%	0%	0%	0%	0%	0%
Disorientation/Recall	0%	0%	0%	0%	0%	0%	13%	5%	0%	6%	0%	0%	0%	0%	0%
Eldercare Staff	0%	0%	0%	0%	0%	0%	0%	5%	0%	3%	0%	0%	0%	0%	0%
Personal Care of Patient	0%	0%	0%	0%	0%	0%	0%	5%	0%	3%	0%	0%	0%	0%	0%
Total	3	13	35	4	55	3	8	19	2	32	1	2	6	0	9
	**Primary Negatives**					**Secondary Negatives**					**Tertiary Negatives**				
	The person I cared for was _____ at the time I started					The person I cared for was _____ at the time I started					The person I cared for was _____ at the time I started				
**Tags**	**26-35**	**36-55**	**55-75**	**75-95**	**Total**	**26-35**	**36-55**	**55-75**	**75-95**	**Total**	**26-35**	**36-55**	**55-75**	**75-95**	**Total**
Personal Care of Patient	20%	31%	22%	40%	25%	25%	20%	5%	0%	11%	0%	17%	0%	0%	5%
Communication with Patient	20%	15%	22%	20%	20%	25%	10%	20%	0%	17%	0%	17%	0%	0%	5%
Emotional Struggle/Conflict	20%	8%	22%	0%	16%	25%	20%	15%	50%	19%	50%	33%	8%	0%	20%
Eldercare Staff	0%	0%	13%	0%	7%	0%	0%	5%	0%	3%	50%	0%	17%	0%	15%
Healthcare	0%	23%	0%	0%	5%	0%	0%	5%	0%	3%	0%	0%	8%	0%	5%
Isolation	20%	8%	3%	0%	5%	0%	0%	0%	0%	0%	0%	0%	0%	0%	0%
Personal Health	0%	0%	3%	40%	5%	0%	0%	0%	0%	0%	0%	0%	17%	0%	10%

Access to Resources	0%	8%	3%	0%	4%	0%	20%	5%	50%	11%	0%	0%	8%	0%	5%
Safety	0%	0%	6%	0%	4%	0%	0%	5%	0%	3%	0%	17%	8%	0%	10%
Disorientation/Recall	0%	0%	3%	0%	2%	0%	10%	10%	0%	8%	0%	0%	0%	0%	0%
Family	20%	0%	0%	0%	2%	0%	0%	5%	0%	3%	0%	0%	0%	0%	0%
Family Responsibilities	0%	8%	0%	0%	2%	0%	0%	0%	0%	0%	0%	0%	8%	0%	5%
Longterm Care	0%	0%	3%	0%	2%	25%	10%	5%	0%	8%	0%	0%	8%	0%	5%
Daily Routines	0%	0%	0%	0%	0%	0%	0%	10%	0%	6%	0%	0%	8%	0%	5%
Finances	0%	0%	0%	0%	0%	0%	0%	10%	0%	6%	0%	17%	8%	0%	10%
Work Performance	0%	0%	0%	0%	0%	0%	10%	0%	0%	3%	0%	0%	0%	0%	0%
Total	5	13	32	5	55	4	10	20	2	36	2	6	12	0	20

	Primary Positives<br>The person I cared for was _____ at the time I started					Secondary Solutions<br>The person I cared for was _____ at the time I started					Tertiary Solutions<br>The person I cared for was _____ at the time I started				
**Tags**	**26-35**	**36-55**	**55-75**	**75-95**	**Total**	**26-35**	**36-55**	**55-75**	**75-95**	**Total**	**26-35**	**36-55**	**55-75**	**75-95**	**Total**
Access to Resources	0%	25%	17%	0%	17%	50%	25%	38%	33%	36%	0%	33%	15%	100%	23%
Healthcare	0%	0%	17%	33%	13%	0%	0%	8%	0%	5%	0%	17%	23%	0%	18%
Support Groups	0%	17%	11%	33%	13%	0%	13%	0%	0%	3%	50%	0%	0%	0%	5%
Finances	0%	8%	11%	33%	11%	0%	38%	8%	0%	13%	0%	0%	0%	0%	0%
Eldercare Staff	0%	8%	6%	0%	6%	50%	0%	8%	0%	8%	0%	0%	0%	0%	0%
Monitors/Sensors	0%	0%	8%	0%	6%	0%	0%	0%	0%	0%	0%	0%	0%	0%	0%
No Support	0%	17%	3%	0%	6%	0%	0%	0%	0%	0%	0%	0%	0%	0%	0%
Communication with Patient	0%	8%	3%	0%	4%	0%	0%	0%	0%	0%	0%	0%	0%	0%	0%
Family	0%	0%	6%	0%	4%	0%	0%	0%	0%	0%	0%	0%	0%	0%	0%
Media	0%	0%	6%	0%	4%	0%	0%	8%	0%	5%	0%	0%	0%	0%	0%
Mobility Devices	0%	0%	6%	0%	4%	0%	0%	0%	0%	0%	0%	0%	0%	0%	0%
Socializing with Peers	50%	8%	0%	0%	4%	0%	0%	0%	0%	0%	0%	0%	0%	0%	0%
Legal/Financial Advisors	0%	8%	0%	0%	2%	0%	0%	0%	0%	0%	0%	0%	0%	0%	0%
Longterm Care	0%	0%	3%	0%	2%	0%	0%	8%	33%	8%	0%	17%	8%	0%	9%
Social Workers	50%	0%	0%	0%	2%	0%	0%	0%	0%	0%	0%	0%	0%	0%	0%
Tech not Helpful	0%	0%	3%	0%	2%	0%	0%	0%	0%	0%	0%	0%	0%	0%	0%
Therapist/Counselor	0%	0%	3%	0%	2%	0%	0%	0%	0%	0%	0%	0%	8%	0%	5%
Adovacy Organizations	0%	0%	0%	0%	0%	0%	0%	4%	33%	5%	0%	0%	8%	0%	5%
Trackers	0%	0%	0%	0%	0%	0%	0%	8%	0%	5%	0%	0%	0%	0%	0%
Personal Care of Patient	0%	0%	0%	0%	0%	0%	0%	4%	0%	3%	0%	0%	8%	0%	5%

Personal Health	0%	0%	0%	0%	0%	0%	13%	0%	0%	3%	0%	17%	0%	0%	5%
Emotional Struggle/Conflict	0%	0%	0%	0%	0%	0%	0%	4%	0%	3%	0%	0%	0%	0%	0%
Isolation	0%	0%	0%	0%	0%	0%	13%	0%	0%	3%	0%	0%	0%	0%	0%
Telehealth	0%	0%	0%	0%	0%	0%	0%	4%	0%	3%	0%	0%	8%	0%	5%
Religious Experiences	0%	0%	0%	0%	0%	0%	0%	0%	0%	0%	0%	0%	8%	0%	5%
Safety	0%	0%	0%	0%	0%	0%	0%	0%	0%	0%	0%	0%	8%	0%	5%
Legal/Financial Advisors	0%	0%	0%	0%	0%	0%	0%	0%	0%	0%	50%	0%	0%	0%	5%
Total	2	12	36	3	53	2	8	26	3	39	2	6	13	1	22

Table 3. Coding of Survey Narratives by Relation to PLWD

	Primary Positives					Secondary Positives					Tertiary Positives				
	Relation of PLWD					Relation of PLWD					Relation of PLWD				
Tags	Father/FIL	Grandparent	Mother/MIL	Spouse	Total	Father/FIL	Grandparent	Mother/MIL	Spouse	Total	Father/FIL	Grandparent	Mother/MIL	Spouse	Total
Expressions of Gratitude/Connection	56%	0%	36%	43%	43%	33%	100%	53%	0%	46%	0%	0%	0%	50%	11%
Rememberance	22%	100%	18%	21%	21%	0%	0%	7%	0%	4%	33%	0%	0%	0%	11%
Being with Children	0%	0%	9%	8%	8%	0%	0%	0%	0%	0%	0%	0%	0%	0%	0%
Family/Social Events	0%	0%	9%	8%	8%	33%	0%	0%	17%	13%	33%	0%	0%	0%	11%
Music/Dance	0%	0%	9%	6%	6%	0%	0%	7%	0%	4%	0%	0%	0%	0%	0%
Daily Routines	0%	0%	9%	4%	4%	17%	0%	7%	0%	8%	33%	0%	75%	0%	44%
Art	11%	0%	0%	2%	2%	0%	0%	0%	0%	0%	0%	0%	0%	0%	11%
Being In Nature	0%	0%	5%	2%	2%	0%	0%	0%	0%	0%	0%	0%	0%	0%	0%
Healthcare	0%	0%	0%	2%	2%	0%	0%	0%	0%	0%	0%	0%	0%	0%	0%
Religious Experiences	0%	0%	5%	2%	2%	0%	0%	0%	0%	0%	0%	0%	25%	0%	11%
Spectator Events/Shows	11%	0%	0%	2%	2%	0%	0%	7%	0%	4%	0%	0%	0%	0%	0%
Travels	0%	0%	0%	2%	2%	0%	0%	0%	0%	0%	0%	0%	0%	0%	0%
Disorientation/Recall	0%	0%	0%	0%	0%	17%	0%	7%	0%	8%	0%	0%	0%	0%	0%
Personal Care of Patient	0%	0%	0%	0%	0%	0%	0%	7%	17%	8%	0%	0%	0%	0%	0%
Rare Expressions/Speech	0%	0%	0%	0%	0%	0%	0%	7%	0%	4%	0%	0%	0%	0%	0%
Total	9	2	22	20	53	6	1	15	2	24	3	0	4	2	9

	Primary Negatives					Secondary Negatives					Tertiary Negatives				
	Relation of PLWD					Relation of PLWD					Relation of PLWD				
Tags	Father/FIL	Grandparent	Mother/MIL	Spouse	Total	Father/FIL	Grandparent	Mother/MIL	Spouse	Total	Father/FIL	Grandparent	Mother/MIL	Spouse	Total
Personal Care of Patient	13%	20%	30%	30%	26%	17%	25%	13%	0%	12%	0%	0%	11%	0%	5%
Communication with Patient	25%	20%	20%	15%	19%	33%	0%	20%	11%	18%	0%	0%	11%	0%	5%
Emotional Struggle/Conflict	25%	0%	20%	15%	17%	0%	25%	20%	22%	18%	25%	0%	22%	25%	20%
Eldercare Staff	0%	0%	5%	15%	8%	0%	0%	0%	11%	3%	0%	67%	11%	0%	15%
Healthcare	13%	20%	10%	0%	8%	0%	0%	7%	0%	3%	0%	0%	0%	25%	5%
Isolation	13%	0%	5%	5%	6%	0%	0%	7%	0%	3%	0%	0%	0%	0%	0%
Personal Health	0%	0%	0%	15%	6%	0%	0%	0%	0%	0%	0%	0%	11%	0%	5%
Access to Resources	13%	0%	5%	0%	4%	17%	25%	7%	22%	15%	0%	0%	11%	0%	5%
Safety	0%	20%	5%	0%	4%	0%	0%	0%	11%	3%	0%	0%	22%	0%	10%
Disorientation/Recall	0%	0%	0%	5%	2%	0%	0%	7%	11%	6%	0%	0%	0%	0%	0%
Family	0%	20%	0%	0%	2%	0%	0%	0%	0%	0%	0%	0%	0%	0%	0%
Family Responsibilies	0%	0%	0%	0%	0%	0%	0%	0%	0%	0%	25%	0%	0%	0%	5%
Longterm Care	0%	0%	0%	0%	0%	0%	25%	13%	0%	9%	0%	0%	0%	25%	5%
Daily Routines	0%	0%	0%	0%	0%	33%	0%	0%	0%	6%	0%	0%	0%	25%	5%
Finances	0%	0%	0%	0%	0%	0%	0%	7%	11%	6%	50%	33%	0%	0%	15%
Total	8	5	20	20	53	6	4	15	9	34	4	3	9	4	20

	Primary Solutions					Secondary Solutions					Tertiary Solutions				
	Relation of PLWD					Relation of PLWD					Relation of PLWD				
Tags	Father/FIL	Grandparent	Mother/MIL	Spouse	Total	Father/FIL	Grandparent	Mother/MIL	Spouse	Total	Father/FIL	Grandparent	Mother/MIL	Spouse	Total
Healthcare	25%	0%	5%	22%	14%	0%	0%	6%	0%	3%	0%	0%	36%	0%	18%
Support Groups	0%	0%	14%	22%	14%	14%	0%	0%	0%	3%	0%	0%	9%	0%	5%
Access to Resources	13%	0%	14%	11%	12%	29%	0%	38%	46%	38%	20%	0%	9%	50%	23%
Finances	13%	0%	9%	17%	12%	0%	0%	31%	0%	14%	0%	0%	0%	0%	0%
Eldercare Staff	0%	0%	9%	6%	6%	29%	0%	0%	8%	8%	0%	0%	0%	0%	0%
Monitors/Sensors	0%	100%	0%	6%	6%	0%	0%	0%	0%	0%	0%	0%	0%	0%	0%

No Support	13%	0%	9%	0%	6%	0%	0%	0%	0%	0%	20%	0%	0%	0%	5%
Communication with Patient	0%	0%	5%	6%	4%	0%	0%	0%	0%	0%	0%	0%	0%	0%	0%
Family	0%	0%	5%	6%	4%	0%	0%	0%	0%	0%	0%	0%	0%	0%	0%
Media	0%	0%	9%	0%	4%	0%	0%	0%	8%	3%	0%	0%	0%	0%	0%
Mobility Devices	0%	0%	5%	6%	4%	0%	0%	0%	0%	0%	0%	0%	0%	0%	0%
Socializing with Peers	13%	0%	5%	0%	4%	0%	0%	0%	0%	0%	0%	0%	0%	0%	0%
Legal/Financial Adisvors	0%	0%	5%	0%	2%	0%	0%	0%	0%	0%	20%	0%	0%	0%	5%
Longterm Care	0%	0%	5%	0%	2%	14%	0%	0%	15%	8%	0%	0%	9%	17%	9%
Social Workers	13%	0%	0%	0%	2%	0%	0%	0%	0%	0%	0%	0%	0%	0%	0%
Tech not helpful	13%	0%	0%	0%	2%	0%	0%	0%	0%	0%	0%	0%	0%	0%	0%
Therapist/Counselor	0%	0%	5%	0%	2%	0%	0%	0%	0%	0%	0%	0%	0%	17%	5%
Advocacy Organizations	0%	0%	0%	0%	0%	0%	0%	0%	15%	5%	20%	0%	0%	0%	5%
Trackers	0%	0%	0%	0%	0%	0%	100%	0%	8%	5%	0%	0%	0%	0%	0%
Emotional Struggle/Conflict	0%	0%	0%	0%	0%	0%	0%	6%	0%	3%	0%	0%	0%	0%	0%
Isolation	0%	0%	0%	0%	0%	14%	0%	0%	0%	3%	0%	0%	0%	0%	0%
Telehealth	0%	0%	0%	0%	0%	0%	0%	6%	0%	3%	0%	0%	0%	17%	5%
Personal Care of Patient	0%	0%	0%	0%	0%	0%	0%	6%	0%	3%	0%	0%	0%	0%	0%
Personal Health	0%	0%	0%	0%	0%	0%	0%	6%	0%	3%	20%	0%	0%	0%	5%
Daily Routines	0%	0%	0%	0%	0%	0%	0%	0%	0%	0%	0%	0%	18%	0%	9%
Religious Experiences	0%	0%	0%	0%	0%	0%	0%	0%	0%	0%	0%	0%	9%	0%	5%
Safety	0%	0%	0%	0%	0%	0%	0%	0%	0%	0%	0%	0%	9%	0%	5%
Total	8	2	22	18	50	7	1	16	13	37	5	0	11	6	22

Appendix 4. Interview Transcript Coding

Table 4. Thematic Coding and Synopsis of Interviews

Position/Role	Interview Synopsis	Primary	Secondary	Tertiary
Spouse Caregiver	[Participant] cared for his wife for over 10 years and she passed away in 2020. In terms of the overarching difficulty he had was in the change of his role from partner to caregiver. All the major life decisions that they used to make together, now had to be made by him alone. The biggest of these was to place her in long term care. Personal care and safety became major issues and the relationship was all caregiver, no partner. He shared that when she was settled into the care home, he could resume being her husband--at least a little bit. He was fortunate to have access to and be able to afford a high quality facility and did not have any complaints about their care. He learned of this place by his local Alz Assoc support group, which he credits for carrying him through the years. He even found his second wife there! He emphasized the need to keep living one's own life while also being a caregiver.	Longterm care	Personal Health	Support Groups
Other Family Caregiver	[Participant] cared for her husband for over 5 years and he passed away in 2018. She is the second wife of [Participant] so they are from the same community and shared similar stories and feelings. [Participant] added that the most challenging part of caring for her husband when he was at home (prior to placement) was the anger and aggression he displayed. It was difficult to manage these behaviors because he was not aware of what he was doing and any application of logic was ineffectual due to the cognitive impairment. It was draining and gave her no personal space. She was having panic attacks and felt guilty for the way she had to "lie" to her husband. She found what worked was redirection and white lies. She learned strategies to manage his behaviors from Alzheimer’s Association resources and training. A few years into the Dx he was moved to long term care, in large part for her own personal well being. Same location as [Participant]’s wife.	Emotional Struggle/conflict	Personal Health	Support Groups

Child Caregiver	[Participant] works as a caregiver at Heart's and Mind's Caregivers and worked as a caregiver for her stepmother. Her biggest challenges include grief (watching a loved one fade away) and keeping them safe (making sure they don't hurt themselves while trying to maintain their autonomy). Grief is a daily battle, and her solution is finding a support system (father and coworkers) and finding peace in religion. She tries to cope with the safety aspect by letting the patients do what they want to go or trying their best to assist them and figure out their needs which is trial and error. Both her father and coworkers provide a good support system where they can lean on each other. The co-workers are especially helpful because everyone has different skills and can help manage the needs of the patient. Additionally, when keeping the patient safe, engaging in person-centered-care and trying to provide experiences that align with who they are is a helpful strategy. Although the strategies work well, there isn't always time to implement them successfully when there are a lot of people to take care of and many crises. It's even difficult at home when you are just stuck with the dementia patient and pray for a solution. While the support systems are also beneficial, there's a negative connotation to grief so it's been hard that some relationships have been cut off because the person does not know how to care for the other who is dealing with grief. You need the right environment to allow yourself to go through the grief.	Emotional Struggle/conflict	Safety	
Spouse Caregiver	She was a caregiver for her husband, but he is not in a residential memory care. So [Participant] has the position as a remote caregiver. Finding the right residential care program and evaluating it was a difficult process. She found a friend who was on a board of a community senior center in a city where her spouse's children reside and she placed him in a residential memory care program there. She had a conflict over if she is abandoning him and how frequently she would be able to visit him, but he only remembers his kids now so she knew it was best for him to be close to them. His friends were a resource that recommended the program. [Participant] talked a lot about how she felt that the people in her support group weren't as resourceful as her. They didn't know where to look for information, where all the resources were, and how to be educated on very important decisions, financial liability, strategies, and how to find good diagnostic doctors. Although [Participant] was resourceful and she googled, researched, and found the resources she needed, she thinks there should be a portal for all dementia related resources.	Personal Care of Patient	Access to Resources	Emotional Struggle/conflict

Spouse Caregiver	[Participant] spoke about how there were overwhelming challenges when you are a dementia caregiver because you have little control over your own time and energy. Then, you have occasional control over the person. She felt like she was constantly juggling things and she just kept trying to push forward. Husband ended up going to an Alzheimer care center locally, and that gave her a break from her caregiving duties. A caregiver would come and help her husband and take him to the center which gave [Participant] space to breathe. She felt that the support groups were very helpful. Additionally, it was difficult that [Participant] has her own health problems to deal with on top of caring for her husband. Again having other people help her out was essential. She loved having visitors and appreciated the help. She feels like there was never enough help, and she was always grateful for more. She never wanted to put him in a nursing home because she didn't want to change his environment too much, she thought she could take better care of him at home.	Longterm care	Personal Health	
Child Caregiver	[Participant] is a caregiver for his single mother and expressed feelings of resentment and sadness surrounding his role as her caregiver. He did not plan on being her primary caregiver, intending to look after her for only a few months. Once there, he realized that her condition was worse than he previously knew of and then the pandemic hit pretty much immediately after he came to stay with her. Due to the pandemic and her worsening condition, he ended up moving in with her. He spoke about feeling uprooted from the life and job he led before moving across the country to act as her caregiver; he was simultaneously balancing working from home, with acting as her caregiver, and trying to manage all of the legal paperwork that came with her diagnosis. [Participant] mentioned constantly struggling with feelings of resentment as the result of having to completely put his life on hold and changing the trajectory he was previously on prior to moving back home. He also spoke of feeling frustrated by the communication barrier her dementia imposes as his mother is no longer able to express what is wrong or if something is bothering her; [Participant] mentioned now having to rely on her body language for any indication of how she is feeling. [Participant] also spoke of how upsetting it is to watch her struggle or be completely unable to do things she was previously capable of doing. She is now unable to enjoy activities she previously found enjoyable and he is regularly devastated by the reminder that she can no longer enjoy the things she once did. When asked about how he addresses these issues, [Participant] expressed that adult daycares were extremely beneficial, as it gave them some separation and allowed him to take a break from constantly looking after her. This also provided him with more support resources which he previously lacked.	Emotional Struggle/conflict	Isolation	Access to Resources

Therapist/Specialist	[Participant] is an occupational therapist and runs her own business supporting clients and families in the early stages of dementia and supports their preparation for what's to come in the following weeks, months, etc. She also provides care for her mother. One of the problems she encounters is that people generally do not understand dementia and think of it only as memory loss (rather than a steady decline in ability), and it can be challenging to educate people (including the patients) about these end of life issues that they do not wish to discuss. In those scenarios, it is best to be blunt and talk about changes in skills, as well as asking families and patients where else they are seeing change in their lives and abilities. In her experience working with patients, the caregiver needs to be on the same side as them and help put them at ease by working toward positive emotions to decrease resistance; she has found that saying "yes" to the patients' requests (even if that "yes" is essentially a "no" rephrased) helps give the patient time for an opinion and provides a chance to guide them in the right direction without using force. She also spoke about how looking for adaptations and tools that work for patients is a constant cycle of trial and error, because they require the patient to be engaged with those tools, which is not always guaranteed on certain days and can warrant even more confusion; she specifically referenced 'Alexa' systems and how the reminders used for her mother to take her medication only ended up scaring and confusing her even more. Such complications increase frustration and exhaustion for the care providers, because they also have to be in touch with technology and be aware of what works and what doesn't. In addition, she also expressed frustration about the lack of funding available for more in-home care providers, training and transportation for those caregivers, scheduling complications, and support for caregivers with their own health problems.	Communication with Patient	Access to Resources	Tech not helpful

Staff at day carre facility	[Participant] is a health and wellness coordinator at a senior center locally, where she provides care management to various dementia clients; additionally, she supports her mother and manages her care. She discussed the constant toll that managing her clients' lives has on her mental health, and how her clients are "borrowing [her] brain" and that it can be extremely overwhelming dealing with everything they are experiencing, be it facts, goals, needs, emotions, etc. Maintaining a home-work balance is difficult, and if you spend all of your social capital at work, you're burnt out by the end of the day and have little energy for your home life and your immediate relationships. She mentioned how COVID was a "blessing in disguise," in that her program shut down and, while it was stressful to ensure all clients were contacted in lockdown, it gave her time to reassess and take time off to put herself first and watch her own future. She emphasized how much patience is required of her, since communication with her clients, their adult children, and clinicians can be extremely frustrating. With clients, it takes a lot of trial and error to reach common understandings, and you have to be very flexible to meet their needs (i.e. matching tone/words, comprehension) and understand where they're coming from. With clients' families, she encounters a lot of elder abuse, as many adult children are overwhelmed and emotionally drained; she thus often has to reorient/educate them and connect them to additional resources, but she also cannot be each person's one-on-one coach. Dealing with clinicians and medical professionals is also challenging, as she often has to slow them down and encourage them to treat their patients as a person and not a case; in these circumstances, when a professional is being patronizing, she will stop looking at the doctor and indicate to the professional that they need to address the patient non-verbally or in a more respectful manner.	Emotional Struggle/con flict	Personal Health	Communicat ion with Patient

Child Caregiver	[Participant] was a direct caregiver for her mother for about a year before her passing, and she discussed her struggles with preparedness for caregiving, the financial burden, interactions with her mother, and maintaining relationships and balance between her work life, single parenthood, and dementia care. She discussed her frustration with not knowing what to expect in terms of what care looks like, since she and her brother had few professional resources available and had to conduct their own research; she turned to online sources (such as NIH), local healthcare facilities, and books to find information, and she and her brother would share articles and notes about their findings and how to interpret complicated medical jargon they did not understand. She also discussed the financial burden of care, since her mother was low-income and she and her brother were not in a position to support her financially; their only option was to split up her care, and they could not afford to hire a competent professional to support them. This also resulted in scheduling complications, as [Participant] works full-time and is a single parent to two children, one of whom she homeschools, and her brother had his own children and other obligations. She shared how communication and understanding between them became extremely frustrating and tense at times, and how guilt and anger often manifested during care and after their mother's passing. Finding a space in a nursing home was also challenging, and they had to keep calling multiple facilities daily in the hopes that a bed opened up or their mother's name was remembered; the only time they were able to secure a space was when her mother broke her wrist. She also discussed the emotional difficulties of not being able to reason with her mother or calm her down when she experienced hallucinations or was confused; her approach was to remain calm and change the conversation, and when none of that worked (which it often didn't, since what worked one day did not another time) she would walk out of the room and take a breather.	Access to Resources	Emotional Struggle/conflict	Family Responisbilities

Other Family Caregiver	[Participant] was a caregiver for her father who had early onset frontotemporal dementia, but he passed away in 2013. The biggest challenge that she faced with her role for her father was that there was a lot of uncertainty with his diagnosis because her family initially thought that he was an alcoholic. Her mom looked on the internet and found a study at a research hospital where she could take him to receive an autopsy and they had determined that he had dementia. She is also currently a caregiver for her aunt who has Parkinson's. She supports her aunt everyday and is constantly trying to figure out what she needs to best support her (including filing tax extensions and helping with legal documents). However, she is experiencing emotional burnout because she is a law student and she commutes long distances. She has support from her family and they are in constant communication with a group-text. Each person is responsible for different tasks. She received help from someone she worked with to recommend and find access to services. She noted that she and her coworker had to do the majority of research on their own to find different care options and food delivery options like Meals on Wheels and Japanese community services in Berkeley that deliver meal services. She discovered which services her aunt preferred through trial and error (her Aunt did not like the food on Meals on Wheels so they became responsible for giving her groceries). [Participant] explains that it would be helpful to have a financial advisor, a tax advisor, an attorney that was pro bono, healthier and tastier meals provided in food programs, physical therapy, better transportation (her Aunt lives in an unincorporated area where different services are not as available to her), and easier access to COVID shots. She describes that the government does not provide enough of a safety-net for seniors and for people in low-income. She says that if it were not for her and her family, her Aunt would likely be unhoused. She discovered that her aunt's care facility did not give her aunt enough social interaction. Her care facility does not communicate effectively, does not effectively record-keep dosages and medications, does not have enough staffing, and it was also unclean. The different care facilities she took her father also had similar issues and she felt that they were often neglectful. She says that her experiences with her dad has helped her be on more of a "lookout" for her aunt as she is staying in these care facilities.	Access to Resources	Eldercare Staff	Family Responsibilities

Child Caregiver	[Participant] is a caregiver for his father who was diagnosed with Alzheimer'sthree years prior. He is the oldest child and he holds most of the responsibility in all aspects. He helps with finances and he is working with a financial advisor. He did the research and had just moved his father into a caregiving home for 2.5 weeks (since the interview). He said that the staff and the place is nice, but they did a poor job assessing his father and were not prepared for someone who has dementia. He chose this caregiving home because it seemed that they would give his father more autonomy and said that he could go on walks on his own. However, they changed their mind in 2 days when they did not communicate to the desk that he had dementia, so they recommended that his father move to memory care. Another challenge he is facing is that he worries and feels anxiety about his father's mental health. He receives support from his family, his girlfriend, doctors, coworkers, and he works with a placement agency. He is also concerned financially. His father is a military vet and he has tried to contact the VA, but it is very difficult to have him become eligible for financial assistance due to time conflicts with work. He wishes for an app that can be a centralized resource for finding different caregiving resources.	Finances	Smart phone apps	Personal Health
Spouse Caregiver	[Participant] was a caregiver for her husband before he passed away. She was responsible for helping him "become independent for as long as possible." She arranged for in-home care when she was concerned about him being lost and because a care facility did not work well (not good enough staff). There was an Alzheimer's support group that helped refer him to a care facility but then COVID struck, so they could not get sufficient caregivers. She was encouraged by a social worker working with her husband's physician to do respite care (temporary care), so she took him in during COVID. She has caregiving experience from other persons in her family who have Parkinson's. She also worked as a nursing assistant. Some challenges she listed were that her conversations with her husband became extremely repetitive. She saw it as her responsibility to be cheerful and she told herself that she was an "actor" in the long-run (she memorized and learned her lines). She wanted to be outside and have conversations with other people but COVID made this difficult. She says that her socioeconomic class and long-term insurance has helped her pay for all of the care. During and after her caregiving role, she received counseling and couple's therapy. She uses Concern to find her current counselor. Something her employer did previously that sort of helped her was having a work/life balance coach. She feels that being physically active and working at her job helps her manage her health and her emotional pain.	Access to Resources	Personal Health	Communication with Patient

Spouse Caregiver	[Participant] was a caregiver for her husband before he passed away. She was responsible for paying the bills, taking care of house repairs, and other responsibilities he could no longer do due to his dementia. She made sure all his needs were met, including physically and mentally. The biggest challenge for her was taking care of him 24/7. She found relief going to work three days a week and sometimes with friends. She also hired caregivers for him. Although, she wishes that they engaged him more. Another big challenge for her was learning to try and not correct him when he would say something wrong. This was difficult for her at first, but she had a connection with a woman in geriatrics who told her of a 6 week caregiving course. She learned ways to communicate with him, and best care for herself. But, she doesn't think she has done well caring for herself since she took her caregiving job very seriously and she preferred her company. She thought about a support group through the Alzheimer's Association, but there were time conflicts. She would have loved having one-on-one counseling that specializes with geriatric dementia or a hospice counselor if she had time. She explains her physical challenges because she spent the most time caring for him instead of herself. She has an understanding manager who allows flexibility and she has met with HR services. She says that it would be helpful if the University set up informal support groups on campus, and that there should be emphasis on the difference in caregiving roles for a spouse vs. a parent. She also says that caregivers do not always look out for support, so it is important that we find them.	Personal Health	Support Groups	Communication with Patient
Child Caregiver	[Participant] provides caregiving to her mother long distance. Her mother stays at home, and she was diagnosed the ear prior for early onset dementia and encephalopathy. [Participant] takes over executive function responsibilities like paying bills, meeting with financial advisors, setting up automated bill payments, bank accounts, accessing passwords, and helping with other mental health challenges. The biggest challenge for her as a caregiver is trying to have her mother seek professional help, but [Participant] explains that there is a stigma that prevents her mother from seeking help. Her mother will feel agitated because it can be hard for her to accept and feel dependent on others. [Participant] and her sister will often try to set boundaries and be supportive of their mother even though [Participant] desperately feels the need to "fix things" with her mother. She also thinks that it would be ideal if her mother would live with her, but she also recognizes that her mother needs her own space. [Participant] says that it was difficult to find a therapist through the university's EAP (Employee Assistance program) through a platform called Better Help because therapists are in high demand. She also self-studies caregiving by reading articles, listening to podcasts where they discuss the relationships	Language/Culture	Finances	Personal Health

	between stress and childhood trauma to dementia to help understand her mother, and getting certified for the mental health first aid training.			
Nurse	[Participant] works as a hospice nurse giving end of life care for dementia patients. She expressed concern for the fact that individuals are only eligible for hospice care for six months at a time; however dementia patients exhibit decline in a non-linear fashion and may plateau during their time in hospice but still lose their care eligibility. Because of her role as a hospice nurse, [Participant] spoke about her ability to create connections with the families of dementia patients, which has also made her aware of the shortcomings in the healthcare system. She spoke about the burnout and financial struggles she witnessed the families of patients undergo during the last stage of their loved ones' lives. One of the biggest decisions she sees families grapple with is the decision to medicate the patient. More medication does make the process of caregiving easier and makes the patient more compliant, but also has sedative effects, particularly antipsychotic medication which is often prescribed to address more extreme behavioral issues some dementia patients exhibit. The majority of the problems [Participant] encounters in her work dealt with the healthcare side of this issue. After the sixth month window, patients may be sent back home, which may place additional strain on the caregiver. [Participant] also spoke about the financial aspect of dementia care, saying that she frequently sees caregivers struggle to find the funds to care for their loved ones; she suggested a caregiver stipend in order to address this need as well as other forms of care such as Telehealth and palliative care.	Healthcare	Finances	Access to Resources
Spouse Caregiver	[Participant] cared for his wife who had Alzheimers who died 5 years ago. He expressed his challenges with keeping her clean and moving her from place to place were difficult. These were challenges for him as he wanted to make sure his wife was cared for by preventing falls (when he transported her) and decreasing the potential for other severe illnesses that would complicate her health (by keeping her clean). In terms of transport, some of the solutions he found helpful was getting a low sitting bed, doubling beds, and keeping her at constant altitude to prevent her from being jostled around. In terms of managing her hygiene, nudging her to come take a shower even when she didn't want to at times worked most of the time. He shared that while the physical aspects of her care were difficult and the foreground of his challenges, he shared that he wished that he spent more time with her just being present opposed to somewhat dismissing her as she couldn't really communicate. Dealing with the emotional labor that he faced, he shared that it was important to rely on others and read books to get (as it gave him a broader image of what was happening and gratitude for his wife's demeanor). He also	Personal Care of Patient	Emotional Struggle/con flict	Access to Resources

	mentioned how important it was for him to have an advanced directive especially when his wife deteriorated drastically in the latter parts of her progression.			
Other Family Caregiver	[Participant] cared for both of her parents who had dementia long term. Her unique care experience wasn't as a primary caregiver dealing with the personal care but as a secondary one who dealt with all the logistical stress with getting her parents in long term care. She mentions that the paperwork to get all the support systems in place and where to find and access the resources available to her parents were major obstacles. The reason why she toiled so hard to get the logistics in place was due to the financial struggles that came with affording a good long term care facility. When she eventually found the long term care facility it was emotionally difficult when she had to lie to her parents and see the resistance they put up when going into the facility. She mentions that when she was able to get connected with adult protective services, it was a real watershed moment that opened up all the resources for her parents. She mentions that it was "like a second (at times more stressful) job" where she had to heavily rely on her support system to give her calm and hope for the future. While she got through the process and associated stress, her situation wasn't ideal. She wished that it was simplified and more of it was online. When talking with her brother and the challenges he faced as the primary caregiver, he had a hard time with the wandering. He solves this through making them more independent but through a safe manner by changing the locks to be dementia proof, giving them interactive toys to keep them busy, and knobs off the stove. While the solutions prevented them from injuring themselves in some ways, it took a toll on him as it prevented him from going anywhere.	Finances	Access to Resources	Emotional Struggle/conflict
Child Caregiver	[Participant] cared for her father who was diagnosed 8 years prior for Alzheimer's Disease where she would primarily spend her nights and weekends with him. Her mom was the primary caregiver and she assumed the role as the secondary caregiver, easing off the burden of her mom. She mentions that the biggest challenge she faced was not being knowledgeable and prepared for the symptoms. She mentions that for the initial diagnosis, her sisters didn't believe her and the doctors weren't helpful. This forced her to be the patient's advocate to push for a diagnosis so she could get the resources set up. While she was caring for her father, she mentions the caregiver burnout as she juggled the pressure from work, family and the Alzheimer's situation as she had to give up parts of herself. This was exacerbated with the stress she faced with being in a constant state of flux not knowing what was gonna happen next and it felt like her mind was playing tricks on herself. To combat the caregiver burnout, she found it really helpful to rely on her	Emotional Struggle/conflict	Personal Care of Patient	Support Groups

	network. She talked with friends, attended workshops and support groups in order to not feel alone. When her father was being stubborn, she had to learn how to redirect his attention by lying and to be patient. It was especially important to be PRESENT for the moments that her father would be back to how he was prior given the circumstances. When met with excessive stubbornness and wandering, she would give extra medication as a last resort and that would put him to sleep. This was extremely difficult for her as it didn't align with her mentally and spiritually. She really hopes for medical innovations (drug interventions) for future dementia prevention rather than the mitigation of symptoms.			
